# Influence of self-reported vascular health and physical activity level on intravoxel incoherent motion imaging response to exercise in chronic low back pain

**DOI:** 10.3389/fphys.2026.1925680

**Published:** 2026-10-09

**Authors:** Frederique Dupuis, David B. Berry, Alexia Lau, Lara Havandjian, Zachary Brumm, Anirudh Anand, Armin Zavareh, Samuel R. Ward, Kamshad Raiszadeh, Bahar Shahidi

**Affiliations:** 1 Department of Orthopaedic Surgery, University of California, San Diego, La Jolla, CA, United States; 2 Livara Health, San Diego, CA, United States

**Keywords:** exercise, IVIM, low back pain, magnetic resonance imaging, paraspinal muscles, vascular health

## Abstract

**Introduction:**

In individuals with chronic low back pain (CLBP), degenerative changes in paraspinal muscles, such as fatty infiltration and reduced vascularization, may impair muscles’ physiological response to exercise, limiting recovery potential. Intravoxel incoherent motion (IVIM) imaging enables assessment of muscle microvascular perfusion (D*) and intracellular diffusion (D) in response to exercise. Previous work demonstrated that individuals with chronic LBP exhibit attenuated IVIM responses to exercise, and that these responses predict clinical improvement following rehabilitation. Yet the mechanisms underlying these alterations remain unclear. Vascular comorbidities, frequently reported in individuals with CLBP, may impair exercise-induced vasodilation and limit microvascular perfusion in lumbar muscles, while reduced physical activity (PA) levels, also commonly observed, may contribute to alterations in muscle microstructure. This study investigated whether systemic vascular health and PA level moderate lumbar paraspinal IVIM responses to acute exercise in individuals with CLBP.

**Methods:**

Seventy-nine participants with CLBP underwent lumbar IVIM imaging before and after a lumbar extension exercise. IVIM parameters D, D* and perfusion fraction (f) were measured for paraspinal muscles. Participants were classified as having a known vascular condition (n=26) or no vascular condition (n=53), and stratified into High (n=42), Moderate (n=23), or Low PA (n=14) groups based on the International Physical Activity Questionnaire. Linear mixed models examined the moderating roles of vascular health and PA on IVIM parameters, controlling for age, BMI and relative resistance as between-groups differences were observed.

**Results:**

There was a significant main effect of Vascular condition on D (p=0.01), with the Vascular group having lower absolute values pre and post-exercise. There was a significant Time x Vascular interaction for D* (p=0.02): the Vascular group showed an attenuated response compared to the No vascular group. There was no main effect of PA on IVIM parameters, nor interactions with Time, except a trend for PA main effect on f (p=0.06).

**Discussion:**

Vascular health moderates lumbar paraspinal intracellular diffusion and microvascular responses to acute exercise in CLBP. These findings contribute to a better understanding of the physiological significance of lumbar muscle IVIM parameters in chronic LBP.

## Introduction

Low back pain (LBP) is one of the leading causes of disability worldwide (World Health Organization, 2019) and is frequently associated with structural and functional alterations in the lumbar musculature. Degeneration of the paraspinal muscles, characterized by fatty infiltration (Shahidi et al., 2017b), atrophy (Shahidi et al., 2017b), reduced vascularity (Shahidi et al., 2017a) and fibrosis (Shahidi et al., 2020) has been observed in individuals with chronic LBP. These muscular changes may impact muscles’ functional roles, and potentially lead to reduced physical function (Kokosova et al., 2025), and higher disability (Berry et al., 2019). The mechanisms underlying muscle functional alterations in LBP remain poorly understood, limiting the development of targeted interventions.

Advanced magnetic resonance imaging (MRI) techniques have enabled more detailed and dynamic characterization of muscle tissue beyond conventional structural measures. Among these, intravoxel incoherent motion (IVIM) imaging has emerged as a promising method to assess functional diffusion and microvascular perfusion (Englund et al., 2022b). IVIM distinguishes diffusion of water in the intracellular and extracellular spaces (coefficient D) to diffusion of water within the intravascular spaces (coefficient D*) (Englund et al., 2022b). This technique has been applied in various tissues, including the skeletal muscle (Englund et al., 2022a), where it has shown sensitivity to pathological compositional change (Qi et al., 2008) and physiological stimuli such as acute exercise (Mastropietro et al., 2018; Adelnia et al., 2019; Englund et al., 2022a).

A previous study reported attenuated exercise-induced changes in IVIM parameters in individuals with chronic LBP compared to those without, in both intra and extravascular perfusion parameters (Shahidi et al., 2021). More importantly, the degree of IVIM-induced responses predicted clinical improvement following an exercise-based rehabilitation program (Shahidi et al., 2021), suggesting that altered muscle physiological response to exercise limits recovery potential. IVIM response to exercise shows promise for informing targeted interventions, however, the underlying physiological mechanisms of these alterations require further investigation. Here, we propose two underlying mechanisms for altered IVIM response to exercise.

First, reduced intravascular muscle perfusion as measured with IVIM may be moderated by the presence of systemic vascular impairments, capturing broader aspects of vascular health. Chronic LBP has frequently been associated with vascular comorbidities, including hypertension, high cholesterol, and atherosclerosis (Leino-Arjas et al., 2006; Suri et al., 2023). During exercise, skeletal muscle perfusion increases through vasodilation of the microvasculature, leading to enhanced capillary perfusion and oxygen delivery to active fibers (Saltin et al., 1998; Joyner and Casey, 2015). In the presence of vascular dysfunction, this vasodilatory response is impaired, resulting in reduced muscle blood flow (Dipla et al., 2017; Sayegh et al., 2025). Such alterations may limit oxygen delivery and limit muscles’ adaptation to exercise program.

Second, altered extravascular (i.e., intracellular) diffusion response in paraspinal muscles may reflect local muscle disuse (Englund et al., 2022b), as diffusion (coefficient D) is related to fiber size (Englund et al., 2022b; Berry et al., 2025). Reduced use impacts muscles’ microstructure (Jackman and Kandarian, 2004), leading to fiber atrophy for example, which may moderate IVIM extravascular diffusion measurements (Englund et al., 2022b). Indeed, people living with chronic pain have been reported to exhibit lower physical activity (PA) levels (Parker et al., 2017; Alzahrani et al., 2019; Hanna et al., 2019), which may serve as an indirect indicator of muscle disuse. Thus, the level of PA may be a moderator of muscles’ IVIM responses in individuals with chronic LBP.

The objective of the present study is to investigate whether the IVIM response to acute exercise in lumbar muscles is moderated by vascular health or PA level in people with chronic LBP. We hypothesize that intravascular perfusion (D* and ΔD*), will be attenuated by the presence of a vascular condition. We also hypothesize that intracellular diffusion changes i.e., extravascular diffusion (D), would be attenuated in people presenting lower PA levels, reflecting muscle disuse related atrophy. By assessing these interactions, this study aims to clarify the physiological meaning of altered IVIM-exercise induced changes in the context of chronic LBP and further understand its potential utility as a biomarker.

## Materials and methods

Study sample included participants from 2 different cohorts, recruited from local spine rehabilitation clinics between April 2021 and February 2025 (n=49), and January 2026 and July 2026 (n=34). Participants between 18 and 85 years old who had experienced LBP for more than 3 months and had no contraindications to exercise or MRI were eligible. They were excluded if they had previous back surgery or were being treated for multiple body regions in addition to the lumbar spine. The protocol of this study was approved by the University of California San Diego Review Board (201425). All participants provided written consent prior to their participation in the study, in accordance with the standards of the 1964 Declaration of Helsinki.

### Vascular health and physical activity

At inclusion, all participants completed self-administrated questionnaires using a Research Electronic Data Capture Platform (REDcap) (Harris et al., 2009). Sociodemographic characteristics including age, sex, height, weight and symptoms duration were collected. Patient-reported outcome measures were used to assess symptoms, which included the Oswestry Disability Index (ODI) for LBP-related disability and the Numeric Pain Rating Scale (NPRS) for average pain intensity.

The presence of a known medical condition altering vascular health (vascular condition) was assessed via a self-administrated questionnaire with the following question: “Do you have any physician-diagnosed conditions? If so, please indicate all known diagnosis.” Participants were classified as having a vascular conditions if they reported having been diagnosed by a healthcare professional with coronary heart disease, cerebrovascular disease, high blood pressure (hypertension), or high cholesterol (hypercholesterolemia) as defined by the World Health Organization (2025). The presence of a potential vascular conditions was further verified by documenting any medication taken by participants that is relevant to a cardiovascular condition, and screening physical therapist electronic record.

The PA level was evaluated using the International Physical Activity Questionnaire, long version (IPAQ) (Craig et al., 2003). The IPAQ assesses self-reported PA across multiple domains of daily life: work, transportation, domestic/gardening activities, and leisure-time PA. Participants report the frequency (days/week) and duration (minutes/day) of walking, moderate, and vigorous activities performed in each domain over the previous 7 days. These responses are converted into metabolic equivalent task values (MET), multiplied by the duration of each activity, and summed to estimate total weekly PA, expressed as MET-min per week. Then, people can be classified into one of the three physical activity categories:

- Low: no or some activity is reported, but not enough to meet the moderate or high criteria.
- Moderate: one of the following:
  - 3 or more days of vigorous activity of at least 20 minutes/day, or
  - 5 or more days of moderate activity or walking of at least 30 minutes/day, or
  - 5 or more days of any combination of walking, moderate, or vigorous activity achieving at least 600 MET-minutes/week
- High: One of the following:
  - Vigorous activity on at least 3 days achieving at least 1500 MET-minutes/week, OR
  - 7 or more days of any combination of walking, moderate, or vigorous activity achieving at least 3000 MET-minutes/week (IPAQ Research Committee, 2005).

### Exercise-imaging protocol

All MRI scans were conducted on a 3.0 T system (GE MR750, GE Healthcare, Waukesha, WI, USA) using a CTL phased-array spine coil for signal reception. Participants were positioned supine in a head-first orientation, with the scanner isocenter aligned at the L3/L4 vertebral level. IVIM data were acquired using an axial, multi-slice, two-dimensional diffusion-weighted, spin-echo EPI sequence. Imaging parameters included a field of view of 256 × 256 mm², slice thickness of 8 mm, and 22 slices covering the lumbar spine from L1 to S1. The acquisition matrix was 128 × 128 with TR/TE = 2295/52.5 ms, flip angle = 90°, and spatial-spectral fat suppression. Four signal averages were acquired with diffusion encoding applied along three orthogonal directions. Diffusion weighting was performed using b-values of 0, 10, 20, 40, 70, 110, 160, 220, 300, 400, 500, 600, and 700 s/mm², yielding a total scan time of 347 s (Shahidi et al., 2021).

Diffusion-weighted images were first corrected for distortions and phase artifacts using the TOPUP algorithm (Andersson et al., 2003; Smith et al., 2004), followed by denoising with a principal component analysis–based filter. A two-dimensional median filter (radius of three voxels) was applied for spatial smoothing. The images were then averaged across the three diffusion-encoding directions and the four repeated acquisitions for each b-value. IVIM parameter estimation was performed using a three-stage constrained fitting procedure applied on a slice-wise basis. The signal decay was modeled using the standard biexponential IVIM equation:


$$S\left(\text{b}\right)/{\text{S}}_{0}=\left(1-\text{f}\right)\;e^{–bD}+\text{ f}{\text{e}}^{–bD*}.$$

where S(b)/S0 is the measured signal at each b-value, D the intracellular diffusion coefficient, D* the pseudodiffusion coefficient (related to blood flow), and f the perfusion fraction representing the fractional volume of blood in each voxel. Coefficient D represents intracellular fluid motion (i.e., extravascular diffusion), while coefficient D* represents capillaries blood flow. The approach to solve the IVIM equation on a voxel-by-voxel basis has been previously reported (Englund et al., 2024). Briefly, first the natural log of the high *b*-value data were fit to a first order polynomial to determine *D*. This line was extrapolated to *b* = 0 s/mm^2^ to obtain *f*, and finally the NLLS fit of the full bi-exponential model was used to calculate D*. Fit constraints were held constant for each voxel: *f* = [0–0.5]; D* = [1.5–500] × 10^−3^ s/mm^2^; and *D* = [0–2.5] × 10^−3^ s/mm^2^. If a voxel was fit to the lower or upper limits of the fit constraints, it was omitted from further analysis.

T1-weighted images were acquired with the same field of view, slice thickness, and orientation as the IVIM images. Regions of interest (ROIs) were drawn for the bilateral erector spinae and multifidus muscles combined, to be consistent with previous work (Berry et al., 2018), using Horos (Horosproject, Nimble Co LLC d/b/a Purview, Annapolis, MD, USA), and then applied to the IVIM parameter maps. Data were averaged across left and right paraspinal muscles from L1 to S1.

This imaging protocol was completed before (at rest) and after an acute bout of lumbar extension exercise (post-exercise). The lumbar extension exercise was performed on a Nautilus exercise machine (Nautilus Evo, Nautilus Inc.) with a load corresponding to 60% of a 1-repetition maximum obtained on an isokinetic dynamometer. Participants were asked to perform lumbar extension movement at their own pace, within their comfortable range of motion, until they reached a rate-perceived level of exertion (RPE) of 6/10 (~3 min of exercise) on the Borg CR10. This exercise protocol has been validated to discriminate between exercise intensities (Englund et al., 2022a) and to identify muscle physiological alterations in people with chronic LBP (Shahidi et al., 2021) compared to healthy controls. RPE, exercise duration, resistance relative to body weight, and time to scanner (i.e., time between exercise and post-exercise scan) were recorded.

### Statistical analysis

Participants were classified as having at least one known vascular condition (Vascular group) or no known disease (No vascular group). For PA level, as recommended by the IPAQ criteria, participants were divided into three groups of High, Moderate or Low PA level (IPAQ Research Committee, 2005).

Sociodemographic, low back related-symptoms, PA habits and acute exercise-related characteristics (i.e., resistance used, RPE, exercise duration and time between exercise and post-exercise scan) were compared between vascular and PA groups using independent t-tests and ANOVA, respectively.

The moderator role of vascular condition and PA level on IVIM responses was evaluated using a linear mixed model, with Time (Pre vs Post-exercise), Vascular condition (Vascular vs No Vascular) and PA level (High vs Moderate vs Low) as factors, and participants ID as random intercept. Main effects (Time, Vascular, PA) as well as their interactions (Time x Vascular; Time x PA; and Time x Vascular x PA) were assessed. Inherent *post-hoc* tests, with Bonferroni correction, were conducted to detail effects and interactions. All analysis were conducted using Jamovi software (The Jamovi Project).

## Results

A total of 83 participants were included, however the IPAQ questionnaire was not available for 4 participants in the first cohort. Seventy-nine participants were thus included in the analysis, including 41 women and 38 men, with a mean age of 48 ± 16, and a mean symptom duration of 72 ± 118 months (**Figure 1**). Twenty-six participants had a known vascular condition (n=13 reported high blood pressure, n=16 high cholesterol, n=1 angina, and n=1 pulmonary hypertension, **Supplementary Table S1**). Two reported being untreated, and one participant did not report information on medication. Detailed information is provided in **Supplementary File S1**. The Vascular group was older than the No Vascular group (p<0.01, **Table 1**), but there were no differences in sociodemographic, low back related-symptoms, or PA habits between participants with and without vascular conditions. There was also no difference in exercise duration, perceived level of exertion, or time between exercise and post-exercise scan (p>0.11, **Table 1**). Age was thus included as a covariate, and Age by Time interaction was added in the model.

**Figure 1:**
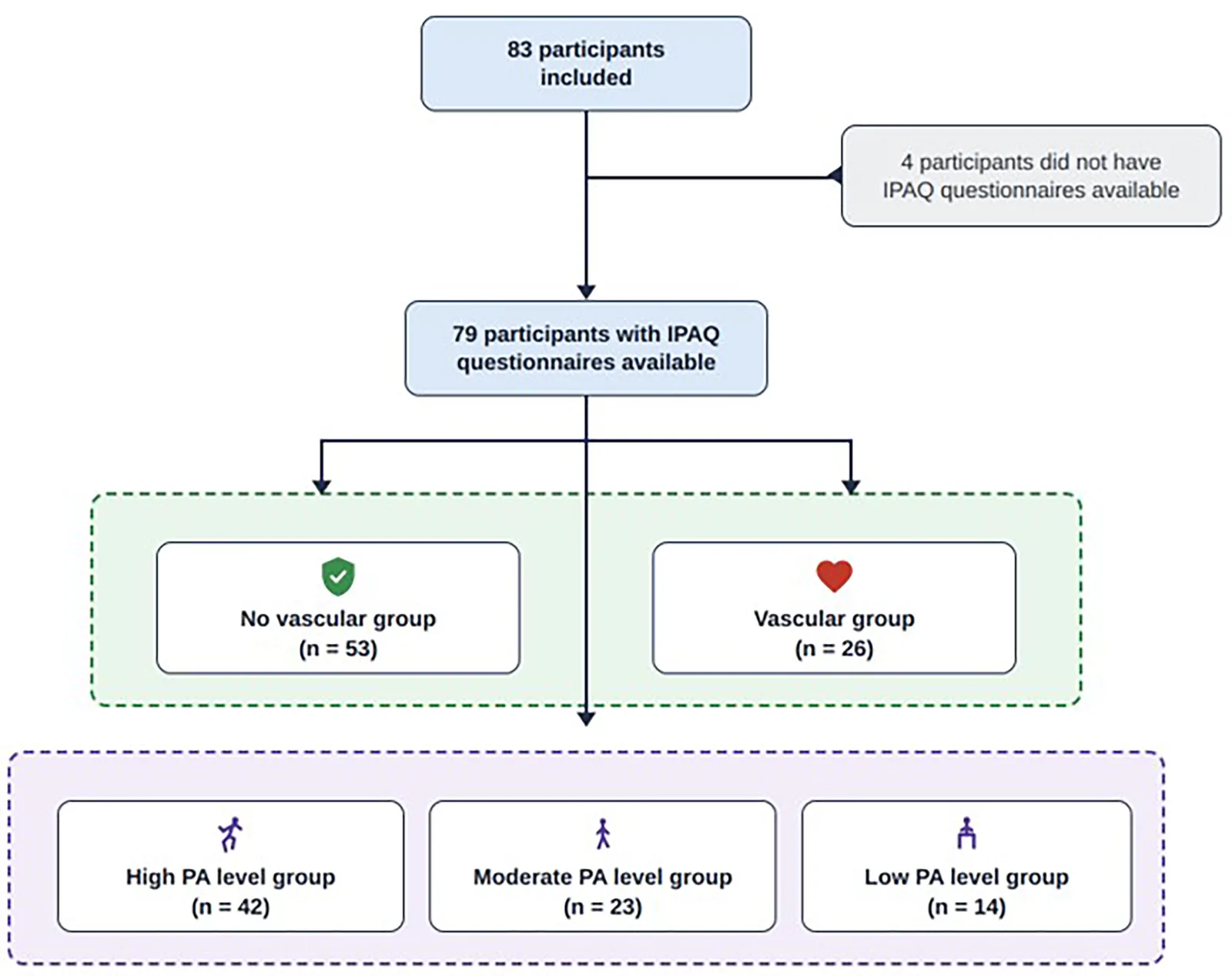
Flow diagram.

**Table 1:** Participant characteristics.

Characteristics	Vascular condition (n=26)	No vascular condition (n=53)	p-values	High PA level (n=42)	Moderate PA level (n=23)	Low PA level (n=14)	P values
Age (years)	**57 (13)**	**43 (16)**	**<0.01**	46 (17)	51 (17)	50 (12)	0.42
Sex (f/m)	13/13	28/25	0.81	23/19	12/11	6/8	0.72
BMI	27 (5)	26 (6)	0.49	**25 (5)**	**28 (5)**	**29 (6)**	**0.02**
Symptoms duration (months)	67 (129)	77 (115)	0.73	74 (109)	56 (81)	95 (182)	0.68
Disability (ODI)	25.8 (8.2)	21.7 (9.9)	0.08	22 (9)	22 (11)	29 (9)	0.08
Pain (NPRS)	3.9 (1.7)	4.0 (1.8)	0.78	3.7 (1.7)	4.1 (1.7)	4.3 (2.1)	0.64
Resistance during exercise (% of body weight)	45 (10)	43 (12)	0.40	**47 (11)**	**41 (12)**	**35 (10)**	**<0.01**
Exercise duration (s)	199 (62)	207 (150)	0.79	175 (44)	251 (90)	231 (210)	0.09
Time between exercise and post-exercise scan (s)	228 (79)	220 (55)	0.64	214 (44)	229 (89)	247 (72)	0.35
RPE (/10)	5.8 (1.4)	6.2 (0.7)	0.11	6.0 (1.0)	6.3 (0.7)	5.8 (1.4)	0.27
MET per week (min)	6103 (6534)	5511 (6345)	0.67	9480 (6641)	1783 (685)	614 (474)	<0.01
PA level groups							
High (n)	13	29	0.33				
Moderate (n)	6	17					
Low (n)	7	7					

Results are presented as mean (SD). PA, Physical activity; BMI, Body Mass Index; ODI, Oswestry Disability index; NPRS, Numeric Pain Rating Scale; MET, Metabolic Equivalent of Task; RPE, Rating of Perceived Exertion. The bold values highlight the significant differences.

Forty-two participants were classified in the High-level PA level group, twenty-three in the Moderate-level group, and fourteen in the Low-level group. There was a BMI difference between groups (p=0.02, **Table 1**), posthoc analysis indicating that the High-level group had significantly lower BMI than the Low-level group (p=0.03). There was also a between-group difference in the resistance used relative to body weight (p<0.01), with the High-level group using a higher resistance relative to their bodyweight than the Low-level group (p<0.01). BMI and resistance relative to body weight were then also entered in the model as covariate, and BMI by Time and Resistance by Time interactions were explored.

### Effect of time and covariates

There was a significant Time effect for f, D, and D* (all p<0.01). There was a significant Time × Age interaction for D (p=0.01) and D* (p=0.03), with Age negatively associated with both outcomes at each time point, indicating that older participants had lower D and D* values overall.

There was a significant Time x Resistance interaction for D (p=0.046), D* (p<0.01), and f (p=0.02). For D*, resistance was positively associated with post-exercise values, indicating that changes in D* in response to exercise scaled with the relative load used. For f, resistance was negatively associated with pre-exercise values. For D, resistance showed a similar non-significant negative trend for pre-exercise and post-exercise values.

BMI also had a significant Time interaction for D (p=0.048), with a negative association with pre-exercise values. No BMI interaction was observed for D* or f.

### Vascular health

There was a significant main effect of Vascular condition on D (p=0.01), with the Vascular group having lower absolute values pre and post-exercise (-9.88e^-5^, p=0.01). There was a significant Time x Vascular interaction for D* (p=0.02), and *post-hoc* contrasts revealed that the Vascular group showed an attenuated response to exercise (-0.0033, p=0.29) compared to the No Vascular group (-0.0062, p<0.01) (**Figure 2**; **Table 2**). Estimated marginal means are provided in **Supplementary Files**, **Supplementary Tables S2** and **S3**.

**Figure 2:**
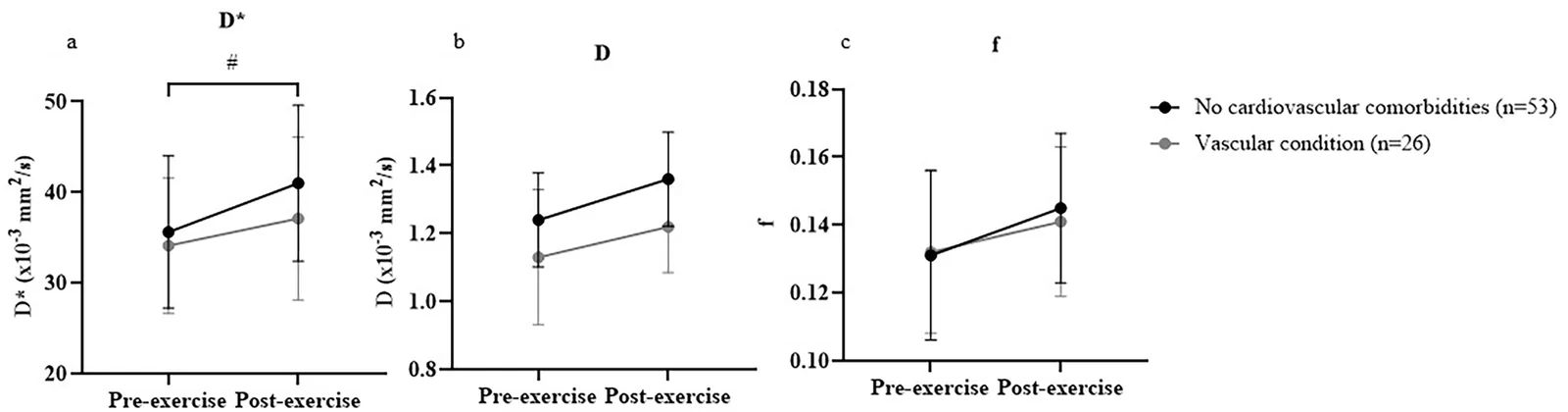
Data are presented as mean (SD). IVIM response and vascular condition. **(a)** D* response: ^#^indicates significant interaction between Time and Vascular condition (p=0.02). **(b)** shows a main effect Vascular condition on D (p=0.01). **(c)** shows f responses, with no significant effects or interactions.

**Table 2:** f, D and D* values pre and post exercise for the vascular and no vascular group.

No vascular condition (n=53)				Vascular condition (n=26)		
Fraction of voxels included						
	Mean	Range	p	Mean	Range	p
Pre	0.74	0.54-0.92	0.33	0.72	0.54-0.91	0.11
Post	0.78	0.57-0.91		0.75	0.59-0.90	
R^2^						
	Mean		Range	Mean		Range
Pre	0.98		0.87-0.99	0.97		0.90-0.99
post	0.98		0.94-0.99	0.98		0.94-0.99
	Mean		SD	Mean		SD
f						
Pre	0.131		0.025	0.132		0.024
Post	0.145		0.022	0.141		0.022
Δ (%)	10.7			6.8		
Estimate	0.013, 95% CI [0.004, 0.021]			0.010, 95% CI [1.38e^-4^, 0.020]		
D (x10^-3^)						
Pre	1.24		0.138	1.13		0.199
Post	1.36		0.138	1.22		0.136
Δ (%)	9.7			8.0		
Estimate	8.04e-5, 95% CI [2.15e^-5^, 1.39e^-4^]			9.05e-5, 95% CI [1.94e^-5^, 1.62e^-4^]		
D* (x10^-3^)						
Pre	35.6		8.4	34.1		7.5
Post	41.0		8.6	37.1		9.0
Δ (%)	15.0*			8.8*		
Estimate	0.006, 95% CI [0.003, 0.010]			0.003, 95% CI [−0.001, 0.008]		

*Significant Time x Vascular interaction for D* (p=0.02). R^2^, goodness of fit.

### PA level

There was no significant main effect of PA level on D* (p=0.60) or D (p=0.37). There was a trend for a PA main effect on f (p=0.07): the Low PA group had lower f values than the High PA group (p=0.06), with no significant difference between the Moderate and High groups. There was no Time x PA interaction for D (p=0.31), D* (p=0.09), or f (p=0.54) (**Figure 3**).

**Figure 3:**
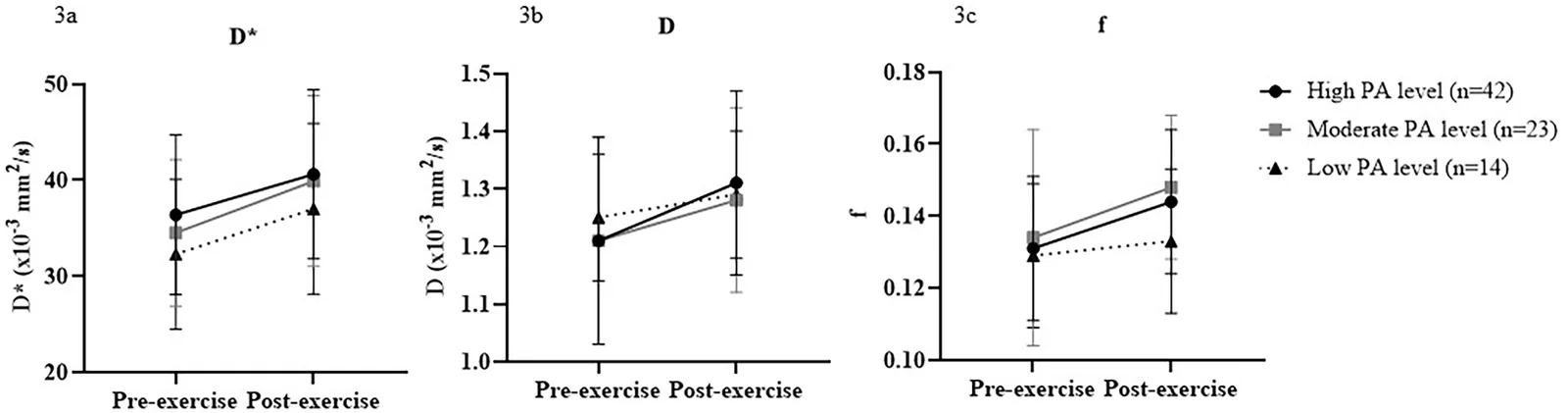
Data are presented as mean (SD). IVIM response and PA level. No significant effect of PA, or interaction was observed between Time and PA. PA, Physical Activity.

### IVIM x vascular health x PA

There was no significant triple interaction between Time x Vascular x PA for D* (p=0.34), D (p=0.41), or f (p=0.62).

## Discussion

This study aimed to investigate whether IVIM response to acute resistance exercise in lumbar muscles is moderated by vascular health or levels of PA as an indicator of muscle disuse in people with chronic LBP. Our first hypothesis was confirmed, as muscle capillary blood flow changes in response to exercise (ΔD*) was attenuated by the presence of a vascular condition. However, contrary to our second hypothesis, no main effect of PA level or interaction with time was observed for intracellular diffusion D.

The current results suggest that reduced pseudodiffusion in response to exercise in the lumbar muscles may be, in part, related to an altered global vascular health. The increase in pseudodiffusion following acute exercise was attenuated in the Vascular group compared to the No vascular group. These findings align with endothelial dysfunction. Under normal physiological conditions, acute exercise induces vasodilatation and increases capillary perfusion to meet metabolic demand (Saltin et al., 1998; Joyner and Casey, 2015), which is measured by the increase in D*. However, vascular conditions such as hypertension and hypercholesterolemia are associated with reduced vasodilatory capacity, which may limit the ability to augment blood flow in the muscles during exercise, as demonstrated by the lack of significant increase in D* (Dipla et al., 2017; Sayegh et al., 2025). This is further supported by evidence of capillary deficit observed in people with chronic LBP when assessing biopsies of the multifidus muscle (Shahidi et al., 2017a), and knowing that capillary rarefaction is influenced by systemic vascular disease (Shono et al., 1999; Robbins et al., 2011; Gueugneau et al., 2016). Our results showed that Age also had an interaction with Time for D*, but because the model already statistically adjusted for Age and Vascular condition, both variables likely explain some variation in ΔD*.

These findings are important because they extend previous evidence that have linked IVIM response to acute exercise to clinical improvement after a resistance exercise-based program for people with chronic LBP (Shahidi et al., 2021). Previous findings suggested that the ability to elicit appropriate microvascular responses to acute exercise is important for clinical improvement, and the present results extend these observations by indicating that such responses may, at least in part, depend on underlying vascular health. Muscle adaptation to exercise typically relies on increased skeletal muscle perfusion to enhance oxygen delivery to active tissues (Saltin et al., 1998; Joyner and Casey, 2015). However, in the context of vascular dysfunction, this vasodilatory response may be blunted, potentially limiting the capacity for physiological adaptation to exercise-based rehabilitation. Additionally, the observed scaling of D* with relative exercise resistance demonstrates that IVIM-response is sensitive to the magnitude of exercise dose delivered. The precise relationships between vascular health, muscle function and potential for recovery following exercise-based rehabilitation warrants further investigation.

Interestingly, we observed an attenuated microvascular muscle response in individuals with vascular conditions, despite most participants being treated with medication. As such, it remains difficult to determine whether this attenuation is related to medication use or to the vascular condition itself. This raises the question of whether these vascular conditions are adequately controlled, or whether pharmacological treatments may influence skeletal muscle microvascular health independent of the underlying pathology, an area that remains largely understudied.

In contrast to our initial hypothesis, no main effect of PA level on D response was observed, nor was there a significant PA x Time interaction. Notably, relative resistance differed significantly across PA strata (High: 47%, Moderate: 41%, Low: 35% of body weight) and was included as a covariate with a significant Time interaction for D in the same model. Once the exercise load actually delivered is accounted for, PA level itself does not appear to further moderate the IVIM response. The coefficient D reflects the total diffusion signal within a voxel, and is commonly attributed to intracellular diffusion in the context of IVIM. In a similar approach to IVIM called diffusion tensor imaging, D is similar to mean diffusivity, which is sensitive to fiber size (Berry et al., 2021; Berry et al., 2025). We initially expected PA to influence intracellular diffusion in muscle as a marker of disuse (i.e., reduced fibers size), but these findings suggest that the relationship may be more complex than initially anticipated, or alternatively, that PA level’s influence on D is already captured through its association with relative exercise resistance used.

Interestingly, we observed a main effect of Vascular condition on D absolute values, both pre and post exercise. The smaller D values have been shown to be associated with smaller fibers, which is known to happen in older adults (Cameron et al., 2023). Because the Vascular group was older, we explored the main effect of Age on D values in the model (p<0.01) and observed that the main Vascular effect was still present (p=0.01). Thus, it appears that the presence of a vascular condition itself influences intracellular diffusion of paraspinal muscles. This may be explained by the fact that people with a vascular condition are often more deconditioned, and therefore have smaller fibers (Hedberg et al., 1989), but this is not supported by our observation of equal PA levels between the two groups. It is also possible that capillary rarefaction (Shono et al., 1999; Robbins et al., 2011; Gueugneau et al., 2016) and muscle ischemia contributes to fiber size loss in the vascular group (Hedberg et al., 1989; Koutakis et al., 2015), despite the same level of PA. However, it should be noted that IVIM inherently is not as sensitive to fiber size changes, due to technical limitations surrounding limited number of diffusion directions, precluding accurate estimate of the diffusion tensor, and limited diffusion times (Δ) with conventional spin-echo imaging (Berry et al., 2021). Therefore, lower D values may be related to other structural differences between groups.

### Limitations

This study has some limitations. It should be acknowledged that the IPAQ questionnaire, despite being one of the best self-reported measurements for PA available, has been reported to only have fair to adequate validity (Craig et al., 2003), and tends to overestimate PA level when compared to the use of accelerometry (Craig et al., 2003; Wanner et al., 2016). Similarly, vascular conditions were self-reported. Although we attempted to verify this information using electronic health records, these records were specific to the participants exercise-based rehabilitation treatment, did not contain their full medical history, and also relied on patient self-reported metrics. Consequently, this approach remains subject to inherent limitations, including recall bias and potential misclassification. Also, PA level was used as an indicator for muscle disuse; however, this approach alone may not adequately reflect this construct. More direct measures, including the assessment of muscle fiber size and tissue quality through biopsy, would allow an objective evaluation of muscle microstructure potentially linked to muscle disuse. Finally, exercise workload was estimated by resistance used relative to body weight, RPE, and exercise duration. However, the exercise was self-paced until 6/10 RPE was reached, to be consistent with previous work and to be relevant to clinical exercise prescriptions (Shahidi et al., 2021; Englund et al., 2022a). It is therefore possible that the number of repetitions performed was different between groups, which would affect total workload to the muscles. There was no difference in exercise-related characteristics between the Vascular and No-Vascular group, but we cannot conclude with certainty on the precise biomechanical (i.e., workload) or physiological (i.e., vascularity) reasons underlying the differences observed between the Vascular and No-Vascular group. Potential underlying mechanisms warrant further investigation.

### Clinical relevance and future directions

These findings suggest that self-reported vascular health may play a meaningful role in modulating muscle intracellular diffusion and microvascular responses to acute exercise in individuals with chronic LBP, which has important implications for rehabilitation strategies. Previous work from our group has shown that acute changes in IVIM parameters (D* and D) in response to exercise are associated with clinical improvement, highlighting their potential relevance as markers of treatment responsiveness. The present results extend this perspective by indicating that such responses may, at least in part, depend on underlying vascular health and microvascular function. However, it is important to recognize that not all individuals with chronic LBP have vascular disease, underscoring the heterogeneity of this population. These findings will support further work towards more individualized approaches to rehabilitation, where vascular health may be considered alongside other factors to better tailor interventions and optimize outcomes.

## Conclusion

This study demonstrates that self-reported vascular health, but not PA levels, moderate lumbar paraspinal IVIM absolute mean diffusion (D) and microvascular (D*) response to acute exercise in individuals with chronic LBP Specifically, individuals with self-reported vascular conditions exhibit lower absolute D values, and an attenuated microvascular response (D*) to acute resistance exercise. This study contributes to a better understanding of the physiological significance of lumbar muscle IVIM parameters in chronic LBP. Further research is warranted to clarify the interplay between vascular health, muscle function, and recovery potential.

## Data Availability

The original contributions presented in the study are included in the article/**Supplementary Material**. Further inquiries can be directed to the corresponding author.
