# Supplementary material for: Influence of self-reported vascular health and physical activity level on intravoxel incoherent motion imaging response to exercise in chronic low back pain
Source: Front Physiol. 2026 Oct 9;17:1925680. doi: 10.3389/fphys.2026.1925680 (PMC13690672; doi:10.3389/fphys.2026.1925680)
Supplement: Supplementary file 1 [file Table1.docx]

**SUPPLEMENTARY MATERIAL**

Table S1. Self-reported medical diagnosis and treatment

| **Participants with Vascular condition** | **Diagnoses** | **Treatment** |
| --- | --- | --- |
| 1 | Hypertension | Lisinopril |
| 2 | Hypertension | Amlodipine |
| 3 | Hypertension | Amlodipine |
| 4 | Hypertension | Clonidine |
| 5 | High cholesterol | Reported as "statin" |
| 6 | Hypertension | Spironolactone |
| 7 | Hypertension | Hydrochlorothiazide |
| 8 | Hypertension | Hydrochlorothiazide |
| 9 | Pulmonary hypertension | Sildenafil/Tadalafil |
| 10 | High cholesterol | Atorvastatin |
| 11 | High cholesterol | Atorvastatin |
| 12 | High cholesterol | Untreated |
| 13 | High cholesterol | Hydrochlorothiazide Atorvastatin |
| 14 | Hypertension  High cholesterol  Prediabetes | Losartan |
| 15 | High cholesterol | Atorvastatin |
| 16 | Hypertension | Lisinopril |
| 17 | High cholesterol | Untreated |
| 18 | Hypertension  High cholesterol  Angina | Acebutolol  Amlodipine  Inclisiran sodium  Isosorbide mononitrate  Ivabradine  Nitroglycerin (PRN) |
| 19 | T2 diabetes  High cholesterol | Metformin  Atorvastatin  Fenofibrate |
| 20 | High cholesterol | Repatha |
| 21 | Hypertension  High cholesterol | Losartan  *Reported HC to be treated with meds, but did not report meds* |
| 22 | High cholesterol | Rosuvastatin |
| 23 | High cholesterol | Rosuvastatin |
| 24 | T1 diabetes  Hypertension  High cholesterol | Lyumjev  Valsartan  Repatha  Amlodipine |
| 25 | High cholesterol | Not reported |
| 26 | Hypertension | Losartan |

Table S2. Time x Vascular for D* - Estimated marginal means

| Time | Group | Per-cell N | Estimated marginal means | SE | 95%CI |
| --- | --- | --- | --- | --- | --- |
| 1 | No Vascular | 53 | 0.0347 | 0.00137 | 0.0320-0.0374 |
| 1 | Vascular | 26 | 0.0348 | 0.00173 | 0.0314-0.0382 |
| 2 | No Vacular | 53 | 0.0409 | 0.00142 | 0.0381-0.0437 |
| 2 | Vascular | 26 | 0.0381 | 0.00173 | 0.0347-0.0416 |

Table S3. Vascular effect for D - Estimated marginal means

| Group | Per-cell N | Estimated marginal means | SE | 95%CI |
| --- | --- | --- | --- | --- |
| No Vascular | 106 | 0.00130 | 2.32e-5 | 0.00125-0.00135 |
| Vascular | 52 | 0.00120 | 2.92e-5 | 0.00114-0.00126 |
